# Impact of a nurse-led empowerment programme on home-based care for preterm infants: A quantitative study

**DOI:** 10.6026/973206300214987

**Published:** 2025-12-15

**Authors:** Marudan Anbalagan, Manoharan Saravanan, Shankar Shanmugam Rajendran, Mangalabharathi Sundaram, Kannan Kasinathan, Anitha M Balachandran, Nirmala Asaithambi

**Affiliations:** 1 Department of Paediatric Nursing, Meenakshi Academy of Higher Education and Research (MAHER) (DU), Chennai & Lecture in Nursing, College of Nursing, Madras Medical College, Chennai, Tamil Nadu, India; 2 Department of Research, Meenakshi Academy of Higher Education and Research MAHER (DU), Chennai, Tamil Nadu, India; 3 Department of Pediatric Nursing, College of Nursing, Madras Medical College, The TN Dr MGR Medical University, Chennai, Tamil Nadu, India; 4 Department of Neonatology, Institute of Obstetrics and Gynaecology and Government Hospital for Women and Children & Madras Medical College, Chennai, Tamil Nadu, India; 5 Department of Child Health Nursing, College of Nursing, Madras Medical College, The TN Dr MGR Medical University, Chennai, Tamil Nadu, India; 6 Department of Neonatology Institute of Obstetrics and Gynaecology and Government Hospital for Women and Children & Madras Medical College, Chennai, Tamil Nadu, India; 7 Department of Obstetrics and Gynaecology, Meenakshi Academy of Higher Education and Research (MAHER) (DU), Chennai & Sooriya School of Nursing, Saligramam, Chennai, Tamil Nadu, India

**Keywords:** Preterm infants, nurse-led intervention, home care, stress reduction, fatigue, social support, neonatal discharge planning

## Abstract

The lack of adequate home-based care for mothers of preterm infants poses significant challenges in maternal and infant health, requiring effective interventions to empower mothers with essential knowledge and support. Therefore, it is of interest to assess the impact of the Nurse-Led Mothers Empowerment Programme (NLMEP) on improving home-based care for mothers of preterm infants. A quasi- experimental pre-test post-test design was used, with 100 mothers selected from a tertiary care hospital in Chennai, India. Results showed significant improvements in maternal knowledge, practices, stress, fatigue and social support. The knowledge score increased from 27.31 to 42.11 (p < 0.001) and 82% of mothers demonstrated adequate knowledge and practice. The lack of adequate home-based care for mothers of preterm infants poses significant challenges in maternal and infant health, requiring effective interventions to empower mothers with essential knowledge and support.

## Background:

Preterm birth, defined as the delivery of a baby before 37 weeks of gestation, continues to be a critical global health challenge with profound implications for neonatal survival and long-term health outcomes [1]. Preterm birth is the leading cause of death among children under five, with millions of preterm births occurring globally each year. In India, the issue is particularly alarming, with a significant number of preterm births annually, accounting for a large proportion of global preterm births [2]. The survival rate of preterm infants has improved due to advancements in neonatal care, especially in neonatal intensive care units (NICUs), but preterm infants are still at high risk of complications such as respiratory distress, infections and developmental delays [3]. These complications are exacerbated by the difficulties that mothers face when transitioning from hospital to home care. Mothers of preterm infants are often unprepared for the challenges they will encounter at home, including managing their infant's health, addressing their own emotional distress and providing proper care to ensure the infant's growth and development. Inadequate education and poor caregiving practices during the early days post-discharge are linked to unfavorable outcomes, such as poor growth and frequent hospital readmissions [4]. The home environment can therefore become a critical factor in the infant's continued health, yet many mothers struggle with the demands of caring for a preterm infant without sufficient support or knowledge [5]. Structured educational interventions led by trained nurses have been shown to improve maternal preparedness and caregiving capabilities significantly [6]. Educating mothers about essential topics like thermal regulation, breastfeeding, hygiene, immunization and recognizing early signs of distress can enhance maternal confidence and ensure safer home care practices. These interventions not only promote infant health but also reduce maternal stress and fatigue, providing a more supportive environment for both mother and infant [7]. In this context, Nurse-Led Mothers Empowerment Programmes (NLMEP) have emerged as a practical approach to enhancing the quality of care that mothers provide to their preterm infants post-discharge [8]. These programs, typically delivered by skilled nurse educators, empower mothers with the knowledge and practical skills needed for the optimal care of their infants [9]. They also offer emotional support, which helps mitigate the anxiety and uncertainty that many mothers face. The present study aims to evaluate the impact of such a program on the caregiving practices of mothers with preterm infants, with the goal to improving home care practices and reducing the likelihood of rehospitalization [10]. Therefore, it is of interest to determine the effectiveness of the Nurse-Led Mothers Empowerment Programme in improving the home care practices of mothers with preterm infants and to provide evidence for the integration of similar programs into standard maternal care practices.

## Methodology:

The study employed a quasi-experimental one-group pre-test post-test design. The study population consisted of 100 primi mothers of preterm infant selected using convenience sampling techniques at a tertiary care teaching hospital in Chennai, India. Mothers were purposively selected based on their ability to comprehend Tamil or English and their willingness to participate in post-discharge follow- up. The intervention the Nurse-Led Mothers Empowerment Programme was implemented by trained nurse educators and consisted of two days of structured education combined with hands-on demonstrations that included guidance on feeding techniques, hygiene, thermal care, immunization, infection prevention and follow-up care. The Data collection tools used in this study included self-structured questionnaire to assess knowledge and practice and standardised tool on the Parental Stress Scale (13 items), the Fatigue Severity Scale (9 items), the Multidimensional Scale of Perceived Social Support (12 items). The Pre-intervention data were collected prior to hospital discharge and post-intervention data were gathered on the seventh day after discharge while they are coming for follow up. All statistical analyses were used SPSS software. Descriptive statistics were used to summarize the participant characteristics and the Paired t-tests were applied to compare mean scores before and after the intervention and Mc Nemar's Chi-square test were used to assess changes in categorical outcomes. Ethical approval for the study was obtained from the Institutional Ethics Committee of Madras Medical College and from the Director of the Institute of Obstetrics and Gynaecology, Egmore, Chennai.

## Results:

Among the 100 preterm babies mothers participated in this study most of the mothers were between 21 and 30 years old and all were homemakers. Seventy-five percent had a normal body mass index. Education wise, 63% had completed higher secondary education and the remaining 37% were graduates. Participants primarily came from semi-urban and rural backgrounds and a majority of them lived in nuclear families. The majority had been married for five years or less. Prior to the intervention, none of the participants possessed adequate knowledge or abilities in preterm baby care. Approximately 47% demonstrated insufficient awareness, while 53% demonstrated a moderate level. High levels of stress and fatigue were frequently observed and most mothers described their perceived social support as moderate. Following the implementation of the NLMEP, all outcome measures improved significantly. Eighty-two percent of the mothers obtained appropriate knowledge and caregiving habits after intervention, with the remaining 18% reaching a moderate level. The average knowledge score increased significantly from 27.31 to 42.11 (p < 0.001) (Figure 1 - see PDF). Among the various domains, the most notable gain was observed in feeding practices, while the smallest improvement was in immunization knowledge. There was also a considerable increase in perceived social support, with mean scores rising from 50.37 to 71.86 (p < 0.001). Table 1 (see PDF) shows Comparison of pretest and posttest mean fatigue score. Mothers reported improvements in emotional support, communication and family involvement. Figure 2 (see PDF) shows comparison of the preterm babies mothers pretest and posttest parental stress score.

## Discussion:

The findings of this study align with previous research that highlights the positive impact of structured, nurse-led educational interventions for mothers of preterm infants. Several studies have explored the effectiveness of similar programs in improving maternal knowledge, caregiving practices and reducing psychological stress and fatigue, all of which were key outcomes in this study. Kujur *et al.*(2022) [11] conducted a study in Odisha, India, where a comprehensive newborn care package program was implemented for postnatal mothers. Like the present study, their findings revealed significant improvements in maternal knowledge and practice. In both studies, the intervention led to an enhanced ability to care for the infants, particularly in areas such as feeding and hygiene practices. This supports the notion that structured education programs are effective in empowering mothers and improving care outcomes for preterm infants. Similarly, Fan *et al.*(2024) [12] evaluated a home-based, post-discharge early intervention program for very preterm infants in China. The study found a significant reduction in parental stress, which mirrors the findings of the current study where maternal stress was significantly reduced post- intervention. The similarity in outcomes suggests that nurse-led programs can be generalized across different cultural contexts, with similar benefits in reducing stress and enhancing caregiving practices. Eduku *et al.*(2024) [13] examined maternal fatigue in the context of preterm infant care and found that educational interventions focused on improving caregiving and social support could alleviate fatigue. This finding is consistent with the results of this study, which showed a significant reduction in fatigue scores after the intervention. The current study provides further evidence that fatigue, an often-overlooked yet crucial factor in maternal well-being, can be effectively addressed through nurse-led education and support. Leahy-Warren *et al.* (2020) [10] explored the role of social support in maternal well-being post-NICU discharge and found that increased social support was associated with lower levels of depressive symptoms and improved caregiving. In this study, the improvement in social support and coping abilities observed post-intervention aligns with the results from Leahy-Warren *et al.*Their study emphasizes the importance of not only providing practical knowledge but also fostering emotional and social support for mothers. This aspect was effectively addressed in the Nurse-Led Mothers Empowerment Programme. The study by Prasad *et al.*(2024) [14] further supports the need for structured educational interventions to improve home-based care for low-birth-weight and preterm infants. Their research found that maternal awareness and practice regarding newborn care improved with targeted education, a finding similar to the results observed in this study. The significant improvement in maternal knowledge and practice in this study, particularly in areas such as feeding and infection prevention, underscores the crucial role of education in ensuring optimal infant care.

## Conclusion:

The Nurse-Led Mothers Empowerment Programme significantly improved maternal knowledge, caregiving skills and psychological well-being. It also enhanced social support and coping abilities. These findings support the inclusion of such interventions in neonatal discharge planning to improve the health and development of preterm infants.

## References

[1] Nabiwemba EL (2013). BMC Public Health..

[2] Blencowe H (2012). Lancet..

[3] Mohini H, Shetty BS (2017). International Journal of Advanced Research..

[4] Liu L (2016). Lancet..

[5] Girotra S (2023). Cureus..

[6] Reddy KM (2022). J Family Med Prim Care..

[7] Guadu T (2025). BMC Pregnancy Childbirth..

[8] Kavitha K (2025). Int J Nurs Stud..

[9] Sanchez-Garcia M (2021). Int J Environ Res Public Health..

[10] Leahy-Warren P (2020). BMC Pregnancy Childbirth.

[11] Minakhi K (2022). Current Pediatric Research International Journal of Pediatrics..

[12] Fan J (2024). BMC Public Health..

[13] Eduku S (2024). Heliyon..

[14] Prasad SNR (2024). Indian Journal of Neonatal Medicine and Research..

